# Supplementary material for: Association of TP53 Alteration with Tissue Specificity and Patient Outcome of IDH1-Mutant Glioma
Source: Cells. 2021 Aug 17;10(8):2116. doi: 10.3390/cells10082116 (PMC8394030; doi:10.3390/cells10082116)
Supplement: Supplementary file 1 [file cells-10-02116-s001.zip › cells-1335464-supplementary/Supplementary Figures.pdf]

Supplementary Information

Association of *TP53* alteration with tissue specificity and patient outcome of *IDH1*-mutant glioma

Balazs Murnyak[1] and L. Eric Huang[1,2]

[1]Department of Neurosurgery, Clinical Neurosciences Center, University of Utah, Salt Lake City, Utah 84132, USA

[2]Department of Oncological Sciences, Huntsman Cancer Institute, University of Utah, Salt Lake City, Utah 84112, USA

Corresponding Author: L. Eric Huang, Department of Neurosurgery, Clinical Neurosciences Center, 175 North Medical Drive East, Salt Lake City, UT 84132, USA.

Phone: +1 801-535-3221

E-mail: eric.huang@hsc.utah.edu

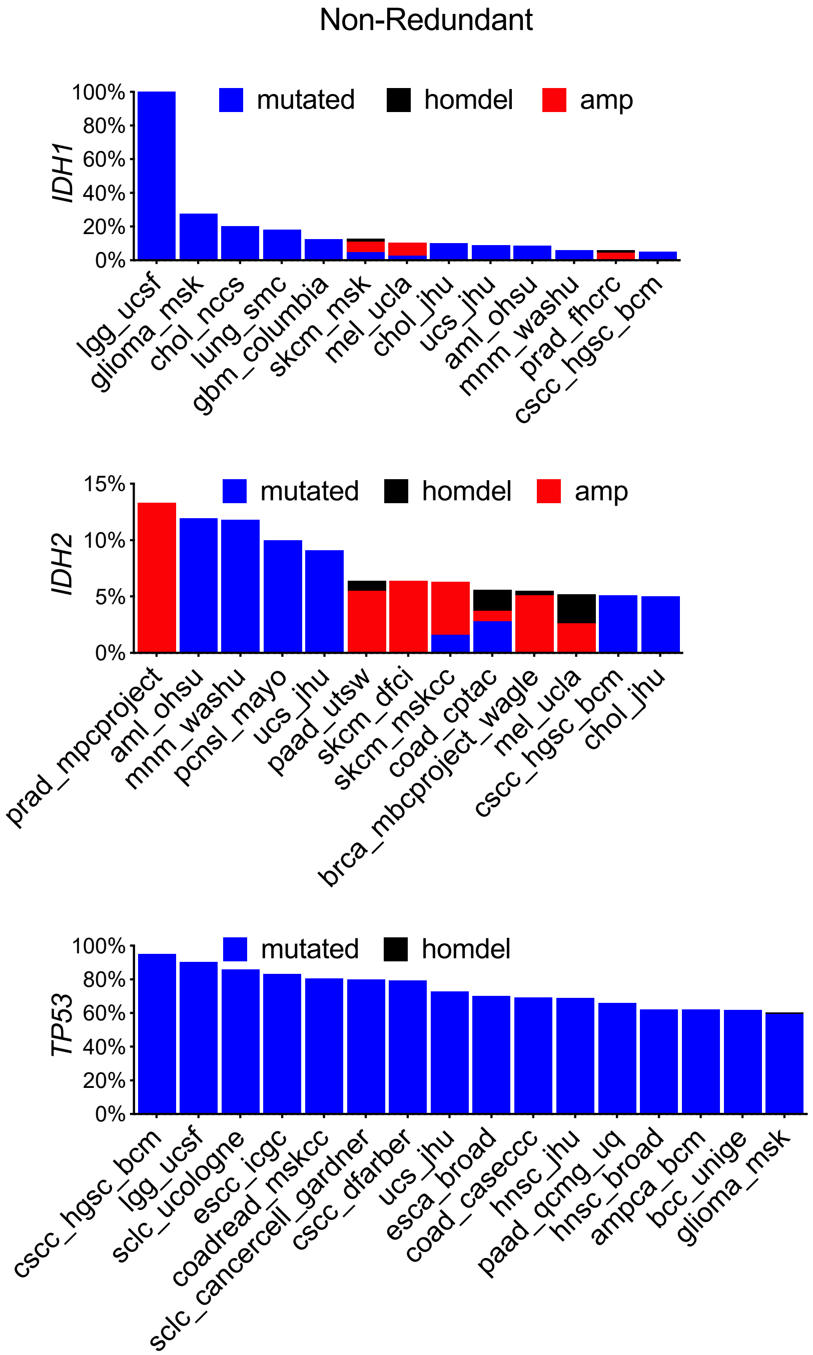

Supplementary Figure S1. Distinctive distribution of *IDH1, IDH2,* and *TP53* alterations in Non-Redundant. Extraction of *IDH1*, *IDH2,* and *TP53* alterations revealed extremely high frequency of *IDH1* mutations exclusively in lower-grade glioma (*A*) in contrast with low frequencies of *IDH2* amplification and mutation (*B*) and high frequencies of *TP53* alterations (*C*) in various cancer types. The cutoff is 5% for *IDH1* and *IDH2* and 60% for *TP53*.

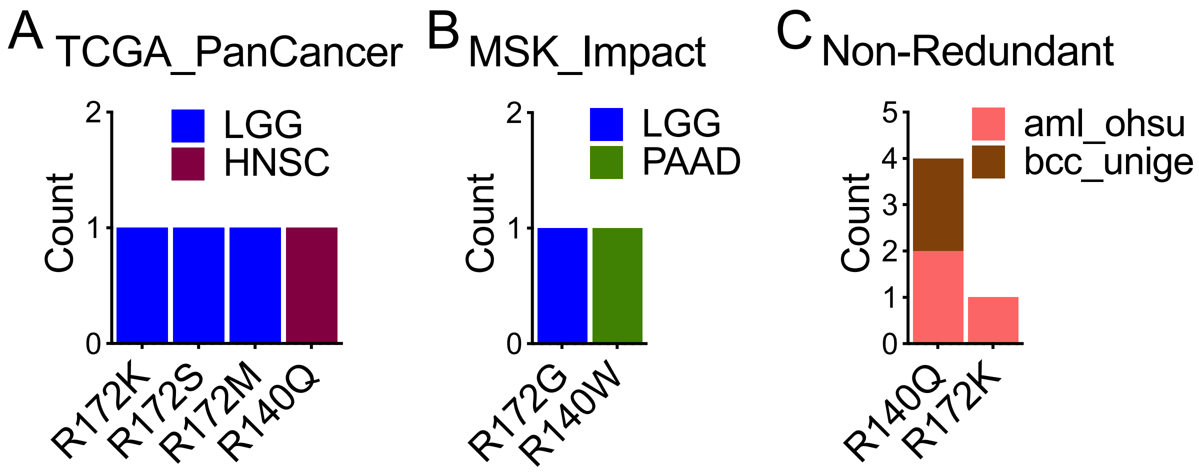

Supplementary Figure S2. Rare co-occurrence of *IDH2* hotspot mutation and *TP53* alteration in human cancer. Cancer types harboring *TP53* alterations are extracted in correspondence to concurrent *IDH2* hotspot mutations from TCGA_PanCancer (*A*), MSK_Impact (*B*), and Non-Redundant (*C*). Sample counts of the cancer types are presented in reference to specific types of *IDH2* mutation.

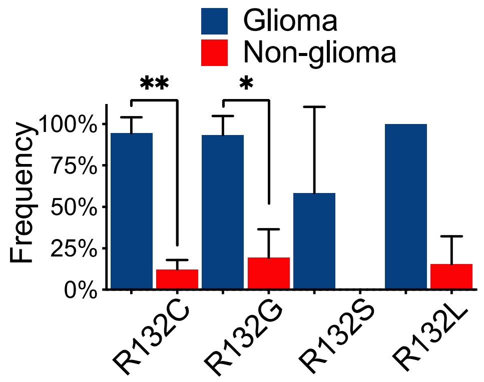

Supplementary Figure S3. Higher frequencies of co-occurrence of specific *IDH1* mutation and *TP53* alteration in glioma. Glioma and non-glioma were compared for their co-occurrence frequencies of specific *IDH1*^{R132X} and *TP53* alteration. *$p < 0.05$; **$p < 0.01$.

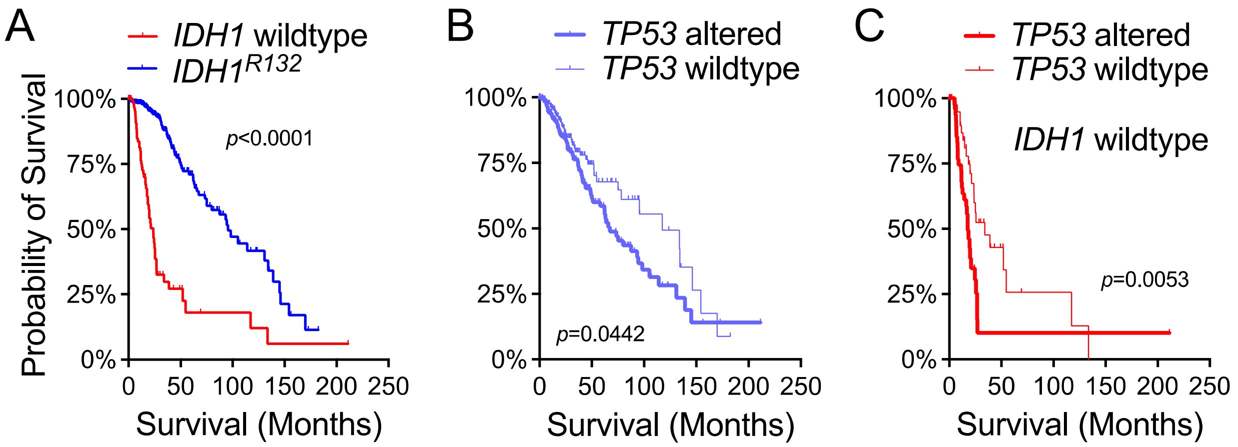

Supplementary Figure S4. *IDH1* status and *TP53* status distinguish patient survival in lower-grade glioma of TCGA_PanCancer. *IDH1* status was analyzed for overall survival (*A*). *TP53* status was analyzed for overall survival (*B*) and in the *IDH1* wildtype subgroup (*C*). Two-tailed *p* values are specified.
